# Oxidative Stress and ROS-Mediated Signaling in Leukemia: Novel Promising Perspectives to Eradicate Chemoresistant Cells in Myeloid Leukemia

**DOI:** 10.3390/ijms22052470

**Published:** 2021-02-28

**Authors:** Silvia Trombetti, Elena Cesaro, Rosa Catapano, Raffaele Sessa, Alessandra Lo Bianco, Paola Izzo, Michela Grosso

**Affiliations:** Department of Molecular Medicine and Medical Biotechnology, University of Naples Federico II, 80131 Naples, Italy; silvia.trombetti@unina.it (S.T.); elena.cesaro2@unina.it (E.C.); rosacatapano92@gmail.com (R.C.); raffaele.sessa@unina.it (R.S.); alessandra.lobianco8@gmail.com (A.L.B.); paola.izzo@unina.it (P.I.)

**Keywords:** acute myeloid leukemia (AML), oxidative stress, ROS, antioxidant systems, ROS-based therapy, chemoreistance

## Abstract

Myeloid leukemic cells are intrinsically under oxidative stress due to impaired reactive oxygen species (ROS) homeostasis, a common signature of several hematological malignancies. The present review focuses on the molecular mechanisms of aberrant ROS production in myeloid leukemia cells as well as on the redox-dependent signaling pathways involved in the leukemogenic process. Finally, the relevance of new chemotherapy options that specifically exert their pharmacological activity by altering the cellular redox imbalance will be discussed as an effective strategy to eradicate chemoresistant cells.

## 1. Introduction

Once considered solely as undesirable by-products of respiration, reactive oxygen species (ROS) are now largely recognized as important signaling molecules. At low levels, under normal and physiological conditions when the balance between ROS production and detoxification is assured, ROS contribute to regulate a large variety of normal cellular functions including proliferation, differentiation, epigenetic modification and quiescence. On the other hand, altered ROS homeostasis has detrimental effects caused by protein, lipid and DNA damage and subsequent disruption of cell functions [1,2]. Therefore, in place of the general term of oxidative stress (OS) comprising all conditions caused by over-burdened and/or disabled antioxidant systems leading to altered redox status, two recent neologisms have been coined to distinguish eustress states as those referring to physiological low oxidative stress conditions involved in redox signaling (ROS^low^ status) from oxidative distress caused by impaired ROS homeostasis leading to disrupted redox signaling and/or oxidative damage to biomolecules (ROS^high^ status) [3]. In this regard, it is also to be underlined that cancer cells depend on moderate increase in ROS levels to promote tumor initiation, development and progression [4]. Thus, it is evident that the tight control of intracellular ROS production and detoxification exhibits fundamental biology relevance either in normal or cancer cells [5]. Acute myeloid leukemia (AML), the most common hematological malignancy in adults, is an aggressive disorder resulting from the clonal expansion of myeloid precursors arrested at different stages of maturation that progressively infiltrate the bone marrow, blood and other tissues [6]. The last four decades have seen great advances in therapeutic options that have positively impacted the overall response rates in AML, especially among younger patients. However, long-term prognosis is still poor with a 5-year survival rate of about 20–25% with a high risk of relapse and development of chemotherapy resistance particularly in older patients [7]. AML is a very heterogeneous disease characterized by complex molecular and cytogenetic abnormalities [8], although some features including altered cellular redox status with high ROS levels are common hallmarks of AML cells [9]. Compelling evidence indicate that impaired redox homeostasis exerts key roles in the leukemogenesis process in hematopoietic stem and progenitor cells that, as a result of successive mutational events, have lost the capacity to control proliferation and to differentiate into mature blood cells, thus giving rise to clones of leukemic stem cells (LSCs), quiescent cells characterized by uncontrolled self-renewal ability and indefinite proliferation potential. Increased redox state correlate with mutational events by promoting the activation of oncogenes, inactivation of tumor suppressor genes, increased aerobic metabolism and mitochondrial dysfunction [10]. These altered pathways generate genomic instability and promote the development and progression of leukemia by up-regulating pathways that sustain cell proliferation, survival, invasion, migration, and metabolic adaptation [11,12]. Notably, in AML cells high ROS levels are compensated for by robust antioxidant systems to avoid excessive ROS production and to protect leukemic cells from oxidative stress-induced cell death [13,14]. Anyway, given their high rate of ROS production, AML cells have a lower buffering capacity against ROS disruption that make them more sensitive to pro-oxidant treatments than their normal counterpart [15].

Accumulating evidence over the past several years has indicated that the high relapse rate in AML results from inability of conventional treatments to efficiently target the LSC population.

Similarly to normal hematopoietic stem cells (HSCs), LSCs exhibit stem cell-like properties, including self-renewal and patterns of undifferentiated hematopoietic cells although molecular mechanisms controlling these processes in LSCs are dysregulated with respect to their normal counterpart [16,17]. Furthermore, HSCs and LSCs significantly differ in their metabolic pathways. In fact, whereas HSCs mainly rely on glycolysis, LSCs depend on oxidative metabolism for their survival [18,19]. As a whole, these disturbed pathways confer drug resistance capabilities on LSCs that make them more resistant to chemotherapies [20,21].Therefore, there is urgent need of identifying novel therapies more effective in targeting quiescent, chemotherapy resistant LSCs to eradicate AML residual disease.

Here we reviewed the most recent knowledge regarding aberrant ROS production and redox state in AML and novel perspectives to target metabolic vulnerabilities of refractory/chemoresistant AML cells to treat acquired resistance and/or disease relapse.

## 2. Energy Metabolism and Cell Redox State in Myeloid Stem Cells

Leukemic cells originate either from multi-potent progenitors (MPPs) or hematopoietic stem cells (HSCs) responsible for life-long production of all blood cells lineages that, as a result of successive mutational events, have lost the capacity to control proliferation and to differentiate into mature blood cells. These events give rise to LSCs clones that have acquired increased proliferative capacity and consequently undergo irregular differentiation, thus generating premature abnormal blood cells [18]. In recent years, accumulating evidence indicates that maintenance of HSC properties including self-renewal, multipotent differentiation potential and relative quiescence requires specialized stem cell niches in the bone marrow microenvironment that play a key role in the control of cell regenerative activities and maintenance of the stem cell pool homeostasis [22,23]. In this regard, according to the evidence that lower levels of intracellular ROS contribute to maintain a quiescent cellular status, HSC are found located in endosteal niches, the most hypoxic regions of the bone marrow where reduced oxygen availability promotes anaerobic metabolism that, in turn, allows maintenance of ROS ^low^ conditions required to sustain stemness and multipotency properties of progenitor cells [16,24]. Therefore, balanced ROS levels ensure a correct hematopoietic process as a ROS ^low^ state participates in maintaining the pluripotency of HSCs whereas an increase in ROS levels accompanies lineage commitment and cell differentiation [16,25]. Consequently, it is clear that lineage differentiation is accompanied by cell migration to different bone marrow niche locations and by metabolism shift towards oxidative phosphorylation and increased ROS production that force HSCs out of quiescence and drive the entry of hematopoietic progenitors into the cell cycle [5]. In this way quiescent, proliferating, and differentiating hematopoietic stem cells display distinct metabolic activities as well as different ROS levels. Signaling pathways involved in hematopoietic differentiation are also regulated by several cytokines, growth factors and other factors that activate ROS-producing pathways in order to modify the expression level of genes controlling HSC self-renewal, proliferation, differentiation or migration within different bone marrow niches [26]. On the other hand, HSCs are highly sensible to oxidative stress conditions and thus unregulated ROS levels contribute to significant alterations in their functions. Therefore, HSCs are largely dependent on ROS homeostasis related both to adapted anaerobic metabolism as well as to enhanced antioxidant defense in order to avoid cell damage and stem cell exhaustion due to high ROS levels [27].

Similarly to HSCs as well as to other types of stem cells, LSCs are located in bone marrow hypoxic regions and exhibit low ROS levels, thus providing further evidence that the susceptibility to oxidative stress is a common feature of either normal or abnormal stem cells [16,28,29]. However, even though a ROS ^low^ state characterizes these two types of cell populations, HSCs and LSCs significantly differ in their metabolic pathways. In fact, whereas HSCs mainly rely on glycolysis, the catabolic pathway that ensures lower ROS levels, LSCs are mostly dependent on mitochondrial oxidative phosphorylation (OXPHOS) system, thus requiring a tighter control of mitochondrial integrity partly relying on mitochondrial clearance (mitophagy), in order to sustain high mitochondrial metabolism and, in the meantime, maintain low oxidative stress conditions. In addition to these metabolic properties, LSCs display other distinctive features, including increased levels of glutathione, high sensitivity to disrupted electron transport chain (ETC) activity, and dependence on amino acid catabolism for oxidative phosphorylation and cell survival [30,31,32]. Thus, it is evident that although LSCs maintain stem cell-like properties and redox patterns similar to their normal counterpart, these disturbed pathways confer drug resistance capabilities on LSCs that make them more resistant to chemotherapies [20,21].

In this context, an interesting contribution was provided by a recent study demonstrating a metabolic shift from amino acid toward fatty acid oxidation to drive oxidative phosphorylation in refractory LSCs isolated from relapsed patients [31,32]. As a whole, these findings highlight, on one hand, the metabolic vulnerabilities of LSCs but, at the same time, the complexity and plasticity of LSCs and the role of metabolic remodeling in adaptive drug resistance as well [17,33,34,35,36].

## 3. ROS Generation and Antioxidant Defense Systems in Myeloid Leukemia Cells

### 3.1. Sources of ROS Production

**Figure 1:**
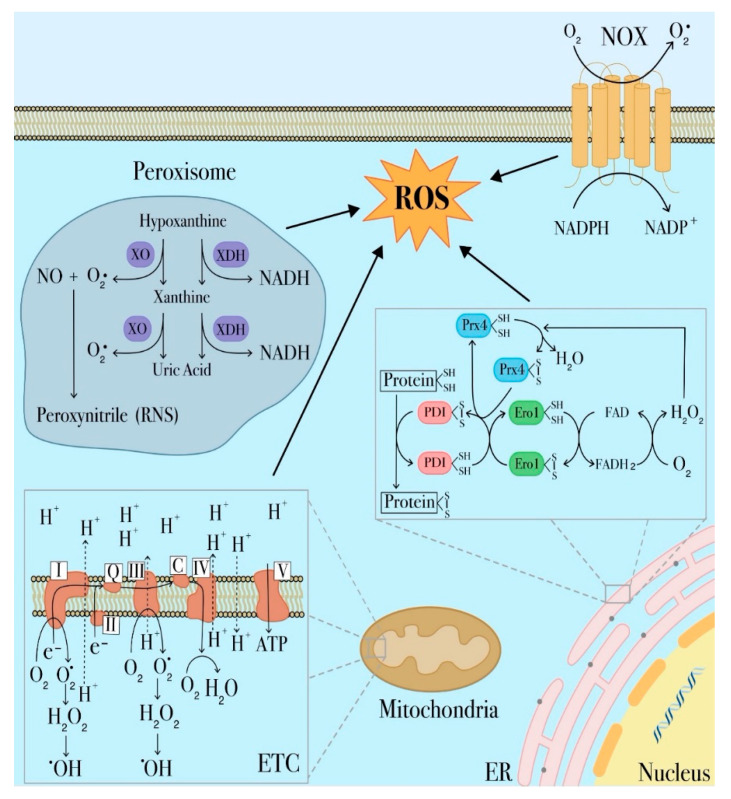
Major sites of reactive oxygen species (ROS) production in leukemia cells. ROS are derived from different cellular compartments and enzymatic systems. The most significant source of ROS in the cell is represented by mitochondria, in which ROS are largely generated by the electron transport chain (ETC). Other ROS-producing mechanisms involve transmembrane NADPH oxidases (NOX), xanthine oxidoreductase in peroxisomes and protein disulfide isomerase (PDI) in endoplasmic reticulum (ER).

There is a large body of literature regarding the sources of elevated ROS levels in leukemic cells [14,37]. Alterations in ROS metabolism are complex and linked to both enhanced ROS production and defective antioxidant defenses. Many metabolic pathways contributing to ROS production such as xanthine oxidoreductase (XOR), uncoupled nitric oxide (NO) synthase (NOS), cytochrome P450 mono-oxygenase (CYP), cyclo-oxygenase (COX) and NADPH oxidase (NOX) activities are altered in myeloid leukemia (Figure 1) [16,38,39]. The NOX family is the first enzyme system reported to produce ROS as a primary function and not just as a by-product of cell metabolism. Several lines of evidence indicate a correlation between NOX-driven ROS formation and leukemogenesis, disease progression and drug resistance [40]. In 2013, Hole et al. observed higher levels of extracellular superoxide in primary AML blasts compared to normal bone marrow samples. Interestingly, cells treated with NOX inhibitors were found to suppress superoxide production more efficiently than electron transport chain inhibitors and mitochondrial ROS scavengers, thus providing evidence that NOX activities were the main source of ROS in these cells [41]. This evidence was further confirmed by additional in vitro studies showing significant reductions in intracellular ROS levels upon NOX inhibition or knockout of NOX isoforms or subunits [16,41]. 

Among cellular organelles, mitochondria are, undoubtedly, a primary source of ROS given their unique role in aerobic metabolism and oxidative phosphorylation. Therefore, as representing the main cause of cellular oxidative stress, dysregulated mitochondrial metabolism plays a prominent role in many leukemia types [42,43,44]. Generation of mitochondrial ROS mainly takes place at complex I and complex III of the electron transport chain (ETC) as a consequence of undesired electron leaks that fail to reach complex IV and univalently react with oxygen to produce the superoxide radical anion (O_2_^−^) (Figure 1) [45]. Superoxide can also be generated during fatty acid oxidation or other mitochondrial oxidoreductase activities, including xanthine oxidase [14,46,47,48]. Superoxide is a membrane impermeable molecule readily dismutated to membrane-diffusible hydrogen peroxide (H_2_O_2_) by superoxide dismutase (SOD) [49]. As a membrane diffusible species, H_2_O_2_ is detoxified to water by catalase, glutathione peroxidase or thioredoxin peroxidase or alternatively can be involved in cell redox signal transduction. Alternatively, H_2_O_2_ can be further reduced to hydroxyl radical (^−^OH), a highly toxic molecule inducing oxidative damage [50,51]. H_2_O_2_ can also be converted in hypochlorous acid (HOCl) by myeloperoxidase (MPO), a member of the heme peroxidase-cyclooxygenase superfamily and a well-known marker of the myeloid lineage that contribute to maintain mitochondrial redox homeostasis [52,53]. Furthermore, it has also been reported that elevated MPO-positive blast cells increase the risk for adverse clinical outcomes in AML and can be related to drug-resistance. In fact, in a recent study, cytarabine-resistant AML cells have been shown to have higher MPO activity and, accordingly, lower levels of mitochondrial and cytosolic ROS levels [54,55,56].

Another important source of ROS in mitochondria is related to the cytochrome catalytic cycle through which a wide range of organic substrates such as lipids, steroid hormones and xenobiotics are metabolized to give rise to superoxide radical and H_2_O_2_ as by-products [14]. Mitochondrial ROS can trigger several redox signaling regulating different physiological processes but, on the other hand, at higher levels they are predicted to cause cell damage and promote apoptotic pathways. It is thus evident that control of redox homeostasis plays a key role since cell fate depends on mitochondrial dynamics and oxidative cell damage: under mild oxidative stress conditions, defective mitochondria are removed through mitophagy in order to reduce ROS generation and sustain cell survival whereas, on the contrary, ROS ^high^ conditions promote mitochondrial fission and dysfunction associated with perturbations in mitochondrial dynamics as so far reported for several pathologies including tumor initiation and progression [14,57]. Deficient mitophagy in AML blasts can also been related to the loss of the autophagy receptor p62 that is normally involved in the control of the number and clearance of dysfunctional mitochondria [58]. Therefore, in accordance with these evidences, myeloid leukemic cells exhibit increased mitochondrial mass associated with enhanced mitochondrial biogenesis and dysregulated mitophagy. A delicate balance is thus required to maintain the proliferative and survival capacities in these leukemia cells that, due to the increased mitochondrial mass and reduced respiratory activity, are more vulnerable to oxidative stress with respect to HSCs and appear to be constantly at the brink of a respiratory system failure [44,59].

Peroxisomes, the major sites of intracellular H_2_O_2_, represent another important source of ROS in leukemia [60] (Figure 1). Several metabolic processes in these organelles generate a wide range of ROS [61]. Two enzymes are responsible for O_2_^−^ generation in peroxisomes, respectively xanthine oxidase (XO) and xanthine dehydrogenase (XDH) [62]. XDH is involved in purine catabolism, by catalyzing the oxidation of hypoxanthine to xanthine and xanthine to uric acid [63,64] and also in the production of reactive nitrogen species (RNS) that are generated when superoxide reacts with nitric oxide produced by NO synthases. The reaction of O_2_^−^ with NO^−^ results in peroxynitrite (ONOO^−^), a highly RNS reactive compound [14]. It is possible that RNS cooperate with ROS in regulating hematopoiesis as well as in driving leukemogenesis in AML, although there is no compelling evidence regarding this role [46]. Although the role of XDH in leukemia is not fully addressed, Zhou et al. found higher XO activity in plasma samples from AML patients as compared to normal controls, and upon disease relapse, an additional elevation in plasmatic XO activity [16,65]. However, further studies are required to better define the role of the xanthine oxidoreductase system in myeloid leukemia [65].

Another organelle responsible for ROS production is the endoplasmic reticulum (ER) responsible for protein synthesis, folding, maturation, and assembly before being exported to the Golgi apparatus, cytosol and plasma membrane. Oxidative protein folding is catalyzed by a number of ER oxidoreductases, among which protein disulfide isomerases (PDI), ERp72, and ERp57. PDI catalyzes thiol-disulfide exchange reactions, which form native disulfide bonds in proteins. During this process, PDI is oxidized by endoplasmic reticulum oxidoreductin-1 (Ero1), which accepts electrons from a reduced PDI and transfers them to oxygen, thereby generating H_2_O_2_ (Figure 1). PDI is also involved in H_2_O_2_ generation through interaction with two members of the NOX family, NOX1 and NOX4, that catalyze the univalent reduction of O_2_ to generate O_2_^−^ and are involved in H_2_O_2_ release in the ER lumen [66,67]. Modifications in these pathways are predicted to lead to unfolding or misfolding of proteins, which in turn can accumulate within the ER lumen resulting in ER stress. This condition disrupts cell homeostasis and initiates the unfolded protein response (UPR), a mechanism used by tumor cells to support their survival and propagation. However, if ER stress is prolonged, UPR triggers tumor cell apoptosis [14,68].

### 3.2. Antioxidant Defences

As previously observed, redox homeostasis is dependent on the balance between ROS production and antioxidant activities. Along with increased ROS production, defective antioxidant defenses in leukemia cells promote increased ROS levels and high oxidative stress status. Indeed, abnormal antioxidant activities contribute to modulate ROS levels and maintain constitutive oxidative stress conditions without surpassing the threshold of an irreparable state of cell injury and activating cell death pathways in different leukemia types [14,69,70]. However, this is still a controversial issue since antioxidant activities in leukemia cells can be either decreased or reinforced depending on specific scenarios. As an example, SOD levels have been found to be up- or down-regulated in different leukemia types. In fact, whereas SOD activities appear to be reduced in ALL, they are up-regulated in AML [71,72,73,74,75].

SODs catalyze the dismutation of O_2_^−^ to form H_2_O_2_ and O_2_ and contribute to protect cells from oxidative stress. However, it is to be noted that, in spite of their potential toxicity, these ROS species may also act as signaling molecules involved in several biological responses including cell proliferation and differentiation [76]. In addition, the presence of specific SOD isoforms in distinct cell compartments indicate that SODs not only act as ROS scavenger and detoxifying enzymes but play crucial roles in ROS homeostasis and ROS signaling between different cell compartments. Furthermore, In AML patients, differences in the expression of SOD in leukemia blasts versus the serum levels of this enzyme have added further complexity to the matter. However, regardless of these apparent discrepancies, SOD expression is functionally required in AML and SOD inhibition lead to enhanced apoptosis in these cells [77,78].

Altered levels of catalase, another important scavenger of H_2_O_2_ mainly located in peroxisomes, have been found in leukemia although with conflicting roles depending on the type of leukemia in which it is expressed and the treatment status [69]. Increased expression or activity of catalase has been observed in AML patient samples [79].

**Figure 2:**
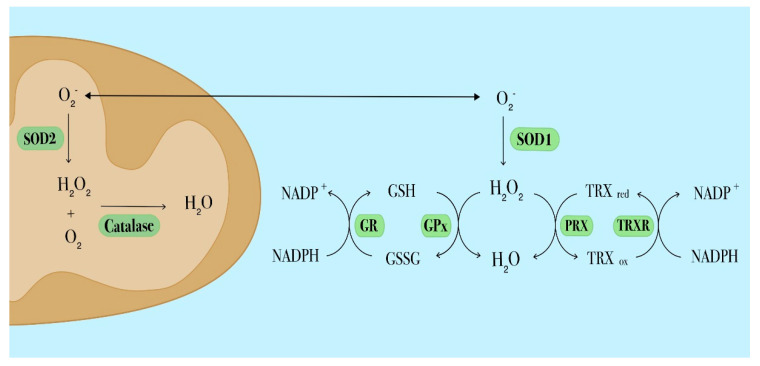
Schematic diagram depicting the main antioxidant systems in leukemia cells. The superoxide dismutase (SOD) catalyzes the dismutation of superoxide into molecular oxygen and hydrogen peroxide, which is then further processed by catalase. Intracellular SOD isoforms have different locations: SOD1 is located in the cytosol, SOD2 in mitochondria. The glutathione (GSH) antioxidant system comprises GSH, glutathione reductase (GR) and glutathione peroxidase (GPx). To perform its antioxidant function, GSH needs to be oxidized into GSSG via GPx. To restore reduced GSH levels, GSSG is converted by GR in a reaction that requires NADPH. The thioredoxin (TRX) antioxidant system involves TRX, peroxiredoxin (PRX) and thioredoxin reductase (TRXR). Reduced TRX catalyzes the reduction of disulfides within PRX. In this process TRX is oxidized (TRXox) and subsequently reduced (TRXred) by thioredoxin reductase (TRXR) through a NADPH-dependent mechanism.

The most abundant non-enzymatic ROS scavenger, glutathione (GSH) and its related enzymatic activities including glutathione reductase (GR), glutathione peroxidase (GPx) and glutathione S-transferase (GST) participate in H2O2 detoxification (Figure 2) and play a critical role in different cellular processes such as proliferation, division and differentiation [80,81]. Alterations of the GSH metabolism have been observed in many cancer types, including hematological malignancies. Recently, Riccio et al. found that enhanced antioxidant capacities due to increased GSH levels and GSH/GSSG ratio in K562 cells, a human chronic myeloid leukemia cell line, were accompanied by the over-expression of GATA-1_S_, the shorter isoform of the transcriptional factor GATA-1.

Interestingly, these findings were consistent with the pro-leukemic role associated to this specific protein isoform [82].

Thioredoxin (TRX) is a redox-active protein with oxygen radical-scavenging and protein-refolding activities in vitro. It is one of the major components of the TRX system, the most important thiol antioxidant network system comprising TRX, TRX reductase (TRXR) and NADPH, (Figure 2). The TRX system is involved in the redox control of proliferation and apoptosis pathways. TRX can also serve as oncogene by conferring growth and survival properties to leukemia cells [83,84]. Over-expressed TRX levels have been found concomitant with increased intracellular ROS levels in relapsed AML and correlate with poor prognosis and a shorter relapse interval [65,85].

## 4. ROS-Mediated Signaling Pathways Involved in the Leukemogenic Process

The variety of cell responses triggered by ROS signaling and leading to the regulation of energy and autophagy pathways, anti-oxidant defenses or stress-responsive signaling cascades generally occur through the regulation of the redox state of few amino acid residues mostly represented by cysteine and methionine. These residues act as redox sensors as they are subjected to redox modifications that can influence protein conformation changes and functions [86]. In this way, redox sensors rapidly respond to changes in ROS levels and activate pathways leading either to cell death or to survival and adaptation to elevated ROS levels. The list of redox signaling proteins is continuously growing and comprises small GTPases, kinases including mammalian target of rapamycin (mTOR), MAP and AKT kinases, and phosphatases such as the family of protein tyrosin phosphatases (PTPs) as well as transcriptional factors and epigenetic modulators that are involved in complex cross-talk networks as recently reported [87,88]. 

**Figure 3:**
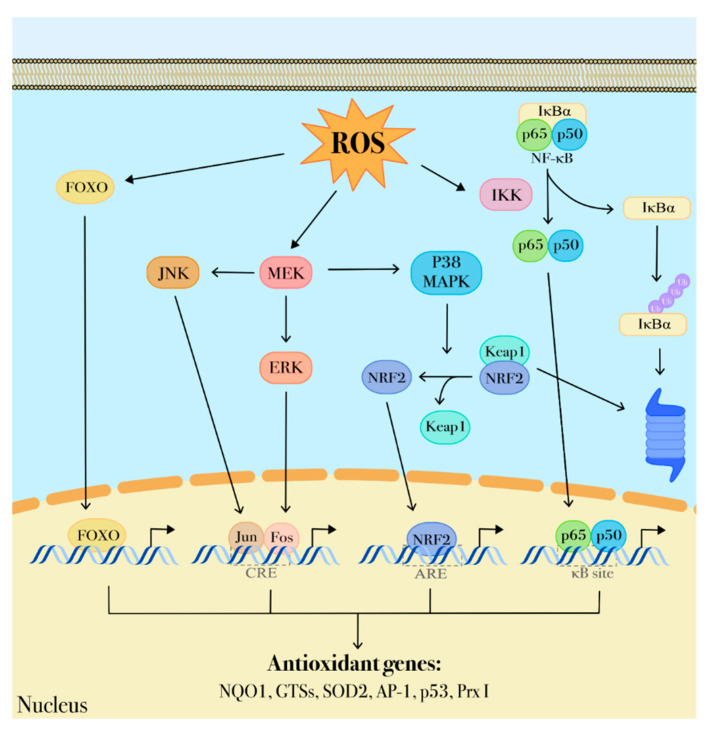
ROS-dependent transduction signaling. ROS are important modulators of intracellular transduction signaling. Through a protein redox-sensor process, ROS can activate the MAPK pathway comprising MEK, ERK, p38 MAPK activities that, in turn, promote nuclear translocation of transcriptional factors including Jun, Fos, NRF2, NF-κB. These factors contribute to regulate several genes involved in antioxidant defenses (p53, NQO1, GSTs, SOD2, Ap-1, p53 and Prx I).

Transcription factors, including members of the forkhead O box (FOXO) family, hypoxia inducible factors (HIFs), helch-like ECH-associated protein (Keap1), Nuclear factor (erythroid-derived)-like 2 (Nrf2), nuclear factor-κB (NF-κB) and the p53 tumor suppressor, have a direct role in redox sensing (Figure 3) since their DNA binding capacity is inhibited by oxidation and, consequently, impaired expression of their target genes has been associated with leukemogenesis and malignant progression [89,90,91]. Similarly, oxidation affects gene expression by impairing the activity of epigenetic modifiers such as some histone deacetylases [92]. One of the mechanisms triggered by oxidative stress signaling is the regulation of nuclear translocation of transcriptional factors that are able to bind antioxidant responsive elements (ARE) and regulate gene expression. In this way, through the transcriptional activation of detoxifying and antioxidant enzymes, they participate in cell response to oxidative stress and coordinate antioxidant activities by protecting non-transcriptional factors that act primarily as ROS sensors. Among these factors there are FOXO family members, also known as longevity factors, that play key roles in stemness maintenance. FOXO factors protect quiescent and stem cells from oxidative stress by upregulating gene expression of detoxifying enzymes including SOD2 and catalase (Figure 3) [93]. In HSCs the protective role of FOXO3 from oxidative stress is also elicited via base excision repair of oxidative DNA damage and regulation of mitochondrial oxidative metabolism [94]. Furthermore, FOXO3 deficiency in HSCs results in inhibition of the ROS-mediated expression of lymphocyte adaptor protein (LNK), an adaptor protein involved in cytokine receptor signaling. LNK depletion leads to constitutively activation of the AKT-mTOR signaling pathway, a central regulator of cellular growth and metabolism, and thus contributes to increased myeloproliferation [95]. In hematopoietic cells FOXO3 is also the major regulator of autophagy and mitophagy that contribute to mitigate oxidative stress by removing damaged mitochondria and toxic proteins [96,97]. Considered together, these findings indicate that, as a common hallmark of several hematological malignancies, impaired activity of FOXO3 leads to mitochondrial disfunctions and aberrant ROS production and thus can be associated with leukemia transformation and progression [98,99].

Nrf2, a common master regulator of the response to oxidative stress, is constitutively expressed in HSCs. Its activity is regulated by Keap1, a redox sensor for Nrf2 signaling that, at low oxidative stress state, targets Nrf2 to ubiquitin-mediated destruction (Figure 3) [100,101]. ROS accumulation promotes Nrf2 release from Keap1 to the nucleus where it forms heterodimers with small Maf proteins and induces the expression of cytoprotective genes and genes involved in critical homeostatic functions [102], such as NAD(P)H quinone oxidoreductase 1 (NQO1), heme oxygenase-1 (HO-1) [103], glutamate-cysteine ligase (GCL) [104], and thioredoxin reductase 1 (TRXR1). However, once malignant transformation has occurred within a cell, Nrf2 acts to support cancer cell survival and to protect from oxidative stress and chemotherapy-induced cytotoxicity through the inhibition of ROS formation. Somatic mutations causing Nrf2 overexpression in cancer cells result in elevated expression of metabolic enzymes which contribute to metabolic reprogramming, thus supporting cell proliferation and transformation [105,106]. Differently from normal hematopoietic cells, Nrf2 is constitutively up-regulated in AML blasts [107] as a result of upstream activation of NF-κB. In particular, NF-κB up-regulates the aberrant Nrf2 expression in vivo through the binding to the κB site on the Nrf2 promoter [108]. NF-κB pathway is a key regulator of different processes including inflammation, antiapoptotic responses, and carcinogenesis and can be influenced by redox-sensitive activation of the PI3K/PTEN/Akt and p38 MAPK pathways that lead to I-κB degradation and subsequent NF-κB activation [41,109]. Some data show that NF-κB activation affects the oncogenic transformation of hematopoietic cells and is required to prevent apoptosis induction in hematopoietic cells expressing BCR-ABL tyrosine kinase that, in turn, triggers ROS production from different cellular sources, including altered mitochondrial activity, NADPH oxidase upregulation and enhanced glucose metabolism [16,110,111]. Thus, NF-κB could play a protective role from apoptosis by suppressing ROS accumulation. In this regard, it is to underline that NF-κB has also many anti-oxidant targets including MnSOD, TRX1, TRX2 and HO-1 (Figure 3) [109]. 

Other redox-responsive transcription factors are the hypoxia-inducible factors, HIF-1α and -2α that regulate gene expression by binding hypoxia-response elements in promoter regions of genes involved in energy metabolism, proliferation, quiescence, and immune function [112]. HIF-1αis highly expressed under hypoxia conditions in HSCs where it promotes stimulation of glycolysis and inhibition of mitochondrial oxidative phosphorylation that, in turn, favors ROS ^low^ conditions and maintenance of HSC quiescence. Although in LSCs HIF-α is activated even under normoxia, its role is still controversial and poorly clarified [113]. In fact, as recently reported, HIF-α control over glycolytic enzymes is not functionally relevant in LSCs with respect to their normal counterpart [114]. Interestingly, it is also emerging that in LSCs HIFs could exert oncogenic activities by promoting autophagy that has a crucial role in regulating cell survival in AML LSCs [115]. Therefore, further studies are required to fully address the role of HIFs in AML and to clarify the processes regulating the interplay between hypoxic signaling and LSC survival.

## 5. ROS-Based Therapies in Myeloid Leukemia Treatment

**Figure 4:**
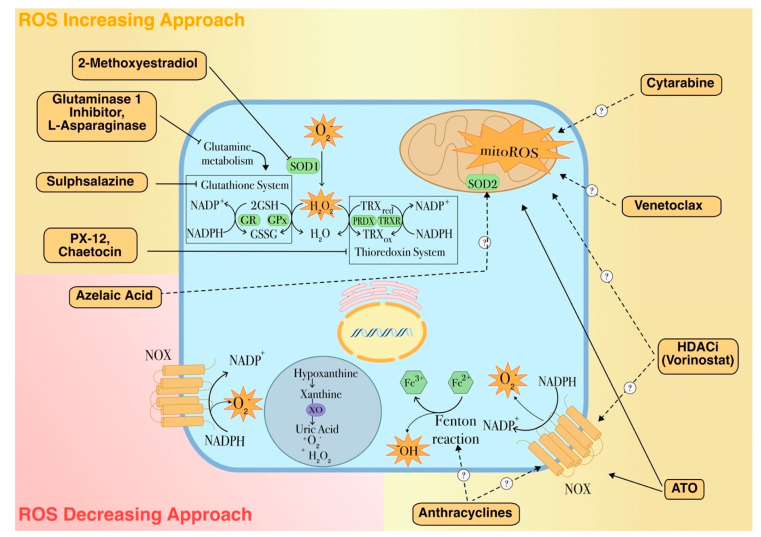
ROS-dependent therapeutic agents for leukemia treatment. The mechanism of action and the molecular targets of pro- or antioxidant drugs used in leukemia are shown. Along with conventional treatments, this figure depicts some promising examples of these approaches. 2-Methoxyestradiol (2ME2), a naturally occurring estrogen metabolite with antiproliferative and antiangiogenic activities is able to induce apoptosis through a ROS-dependent mechanism. Also, this drug is able to target LSCs by inhibiting the transcriptional activity of HIF-1α, that is found over-expressed in LSCs under hypoxic conditions, thus down-regulating pro-leukemic HIF-1α target genes, including the vascular endothelial growth factor (VEGF) [130,131]. L-asparaginase treatment has recently been reported to induce autophagy by promoting apoptosis and cell growth inhibition in AML cells and has synergistic effects with conventional AML chemotherapies [132]. The thiodioxopiperazine natural product chaetocin (SUV39H1 inhibitor) is a competitive substrate and inhibitor of thioredoxin reductase and in this way induces cellular oxidative stress. In addition, as inhibitor of SUV39H1, a co-factor of the transcription factor RUNX1 which has an important role in the regulation of proliferation and self-renewal of hematopoietic stem cells, chaetocin also promotes differentiation of AML cells and has synergistic effects with HDAC inhibitors [133,134]. Dotted arrows with question marks indicate plausible mechanisms of action. Full and dashed arrows indicate well-established or hypothetical molecular mechanisms, respectively.

In recent years, the relevance of the role played by ROS and oxidative state in leukemia has prompt the development of new chemotherapy options that specifically exert their pharmacological activity by altering the cellular redox imbalance (Figure 4). As an instance, in AML a well-established front-line therapy consists of arabinocytosine, a purine analogue that is associated with increased production and release of ROS from mitochondria. The combination treatment with anthracyclines further enhances ROS formation generated as by-products of their own catabolism or produced through a Fenton reaction by interacting with free iron (Figure 4) [69]. In acute promyelocytic leukemia, arsenic trioxide (ATO) has been shown to both increase intracellular ROS production and gene expression of proteins comprising the NOX2 subunit [46]. ATO is also known to directly target the mitochondrial ETC, causing increased ROS production in acute leukemia cells (Figure 4) [69]. 

**Figure 5:**
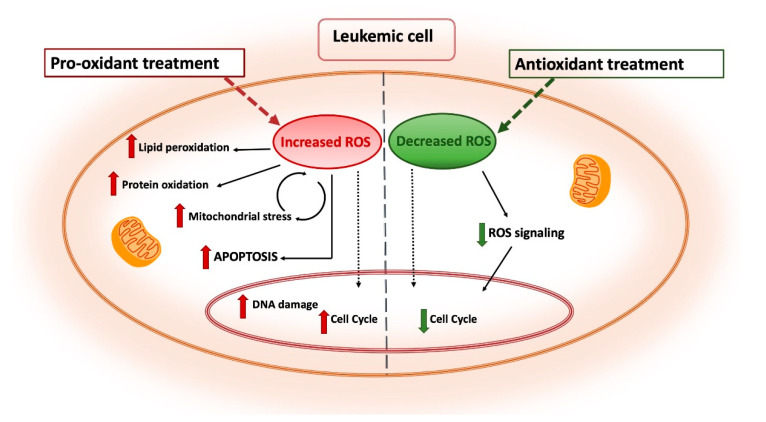
Antioxidant and pro-oxidant strategies as antileukemia therapeutic tools. Pro-oxidant treatments are designed as a strategy to overwhelm the redox adaptation of leukemia cells by inducing oxidative stress incompatible with cellular viability. Enhanced ROS production leads to lipid peroxidation, protein oxidation and DNA damage resulting in increased apoptosis. Conversely, antioxidant treatments are aimed to reduce the leukemogenic potential by tuning down cell proliferation and survival pathways in leukemia cells with high ROS levels. Distinct arrows indicate the involvement of these treatments in different processes and subcellular compartments.

More recently, other pro-oxidant strategies are being developed to modulate ROS levels either by inhibiting antioxidant defenses or by stimulating ROS release [19,116,117]. The pro-oxidant approach is based on the evidence that bulk AML cells are characterized by moderately higher oxidative stress state than their normal counterparts, almost in part related to enhanced antioxidant defenses that could drive drug resistance and confer a competitive advantage to the leukemic clone [15]. However, the accurate fine-tuning between ROS production and scavenging gives leukemia cells a higher sensitivity to external pro-oxidant stimuli that could lead to an unbearable situation of oxidative stress incompatible with cell viability [82]. In line with this concept, Riccio et al. demonstrated that a quercetin pro-oxidant treatment in K562 erythroleukemic cells overexpressing the pro-leukemic transcriptional factor GATA-1_S_ was accompanied by a dramatic depletion in the cellular GSH content and was able to revert the apoptosis resistance shown by untreated cells, thus further supporting the evidence that myeloid leukemia cells are more sensible to external pro-oxidant stimuli [69,82,118]. According to these evidences, several therapeutic strategies are being developed to elevate ROS levels in order to overwhelm the redox adaptation of these cells and induce oxidative stress incompatible with cellular viability (Figure 5) [119]. Recently, Hosseini et al. reported that chemoresistant AML cells have lower ROS levels in response to cytarabine. In these cells, overexpression of myeloperoxidase associated with high rate of mitochondrial metabolism and low redox state was found. Inhibition of myeloperoxidase expression or enzyme activity accompanied by altered mitochondrial redox balance and increased ROS levels was able to restore sensitivity to cytarabine treatment [56].

ROS-based therapies also promise to be an interesting option in eradicating LSC clones. Indeed, in the therapeutic scenario of hematological malignancies, an ever more challenging issue regards the efficient eradication of chemoresistant LSCs populations that are mainly responsible of the high incidence of disease relapse and therapy failure in AML. Therefore, novel treatments specifically and efficiently targeting the reservoir of residual leukemic cell in the LSC niches are an urgent medical need. More recently, transcriptional factors with a direct role in redox sensing have been identified as new promising targets for selectively ablating chemoresistant LSCs cells in AML. Inhibitors of factors that affect cell survival, proliferation and differentiation including histone deacetylases that are known to modulate the activity of p53, and HIF-1α that plays an important role in the self-renewal of hematopoietic stem cells. Also NF-kB is emerged as a therapeutic target to selectively ablate the LSC population sparing the normal counterpart since it is expressed at higher levels only in LSCs [120].

As already discussed, in a similar manner to their normal counterpart, LSCs have low levels of ROS resulting from a combination of low mitochondrial activity and enhanced antioxidant defenses that probably contribute to make these cells less susceptible to increased ROS production induced by conventional chemotherapy. Anyway, differently from HSCs, given the high oxidative phosphorylation LSCs are highly dependent on mitochondrial regulatory pathways to avoid mitochondrial permeability transition or apoptosis [121]. These distinguishing metabolic features could thus represent the Achilles’ heel for specifically targeting and definitively eradicating LSCs [21]. Indeed, in the light of these findings, inhibition of Bcl-2, the master regulator of mitochondrial activity and integrity [122,123], or impairment of mitophagy pathways have been found to affect LSCs survival and to improve response to chemotherapy [9] and could represent a novel strategy for efficient LSC-targeting. In this context, promising results have recently emerged from a clinical trial conducted on older AML patients treated with venetoclax, a Bcl-2 inhibitor, in combination with azacitidine, a hypomethylating agent. This novel approach proved to be more effective in targeting both bulk leukemia and primitive LSC populations as compared to conventional therapies, resulting in striking improved response depth and durability and high remission rate observed even after the first therapy cycle. Interestingly, this treatment also resulted in disrupted energy metabolic pathways leading to reduced oxidative phosphorylation rates specifically achieved through the inhibition of the ETC complex II activity and the induction of ROS production. This study thus provides further proof-of-concept to the importance of mitochondrial oxidative metabolism to sustain survival pathways in AML LSCs and the therapeutic potential of these strategies to efficiently target relapsed/refractory AML cells [124,125]. 

Alternatively, it is also to be noted that, given the role played by ROS in initiation and progression of hematopoietic malignancies, other approaches have been designed to lower ROS levels with the aim to switch off the proliferative signaling in cancer cells [16,46]. This approach is built on the use of agents that inhibit ROS production or function as ROS scavengers (Figure 5). Indeed, whereas pro-oxidant strategies appear to be effective particularly in targeting LSCs and refractory cells, the rationale behind the antioxidant approach is provided by the evidence that circulating AML blasts have elevated ROS levels and reduced antioxidant capacity. Additionally, oxidative stress induced by chemotherapy can ultimately lead to drug resistance and disease relapse. This type of treatment has been proposed to be particularly effective in the treatment of AML patients with mutations in the FMS-like tyrosine kinase 3 (FLT3) that are responsible of elevated ROS levels associated with higher relapse rate. Therefore, ROS-eliminating strategies could represent a promising strategy to treating these forms of AML. Using antioxidants to prevent or treat cancer is not a new concept, in fact a common assumption is that an antioxidant-rich diet might reduce the incidence of leukemia [16]. Antioxidant treatments include supplementation of natural ROS scavengers, or other strategies like the disruption of the ROS-producing mitochondrial electron transport chain that could contribute to the efficacy of chemotherapy drugs since administration of anti-oxidants reduces their cytotoxicity [126,127].

An example of an antioxidant therapeutic approach in AML is provided by azelaic acid (AZA), a natural compound that acts as inhibitor of tyrosinase and other oxidoreductases and ROS scavenger (Figure 4) [128]. It has been shown that AZA treatment suppresses AML cell proliferation and sensitize leukemic cells to chemotherapy by reducing intracellular ROS levels through the up-regulation of antioxidant enzymes such as SOD2 [129].

However, although several studies have documented the benefits of antioxidant drugs in cancer therapies, none has been supported by solid trials performed on a large scale and require further studies [16,119].

## 6. Conclusions

Ever-growing evidence indicate that elevated ROS levels and oxidative stress state play a key role in leukemia onset and progression by stimulating genomic instability, cell survival, growth signaling and drug resistance [33]. Several mechanisms have been described as involved in aberrant ROS production in leukemia cells, including oncogene activation, mitochondrial disfunctions and metabolic changes. Furthermore, many antioxidant systems appear to be dysregulated, leading to increased overall ROS levels that allow cell survival without surpassing a deadly threshold, even under permanent oxidative stress [4,14,15]. On the other hand, ROS levels must be strictly regulated in normal and leukemic stem cells, thus underlining the importance of redox signaling to maintain cell stemness and control hematopoiesis and, in the meantime, unveiling the role of ROS as double-edged swords in leukemia. Therefore, a deeper understanding of the association between oxidative stress and leukemia could provide more insight to support the development of novel ROS-based therapies as a promising antileukemic strategy for eradicating either bulk leukemia cells or LSC populations, particularly in cases of refractory or relapsed diseases.

## Data Availability

No new data were created or analyzed in this study. Data sharing is not applicable to this article.

## Abbreviations

ALL	Acute lymphoblastic leukemia
AML	Acute myeloid leukemia
ARE	Antioxidant responsive elements
ATO	Arsenic trioxide
CLL	Chronic lymphocytic leukemia
CML	Chronic myeloid leukemia
CYP	Cytochrome P450
ER	Endoplasmic reticulum
Ero1	Endoplasmic reticulum oxidoreductin-1
ETC	Electron transport chain
FOXO	Forkhead O box
GCL	Glutamate-cysteine ligase
GPx	Glutathione peroxidase
GR	Glutathione reductase
GSH	Reduced glutathione
GSSG	Oxidized glutathione
GST	Glutathione S-transferases
HDACs	Histone deacetylases
HIFs	Hypoxia inducible factors
HO-1	Heme oxygenase- 1
HSCs	Hematopoietic stem cells
Keap1	Helch-like ECH-associated protein
LNKHSCs	Lymphocyte adaptor proteinHematopoietic stem cells
LSCs	Leukemic stem cells
MPPs	Multi-potent progenitors
mTOR	Mammalian target of rapamycin
NAC	N-acetylcysteine
NF-κB	Nuclear factor-κB
NO	Nitric oxide
NOS	Nitric oxide synthase
NOX	NADPH oxidase
NQO1	NAD(P)H quinone oxidoreductase 1
Nrf2	Nuclear factor (erythroid-derived)-like 2
PDI	Protein disulfide isomerases
PTPs	Protein tyrosin phosphatases
PX-12	1-methylpropyl 2-imidazolyl disulfide
RNS	Reactive nitrogen species
ROS	Reactive oxygen species
SOD	Superoxide dismutase
Tkr1	Thioredoxin reductase 1
Trx	Thioredoxin
UPR	Unfolded protein response
XDH	Xanthine dehydrogenase
XO	Xanthine oxidase
XOR	Xanthine oxidoreductase
